# Protease nexin-1 prevents growth of human B cell lymphoma via inhibition of sonic hedgehog signaling

**DOI:** 10.1038/s41408-018-0063-x

**Published:** 2018-02-26

**Authors:** Xiangke Xin, Yunchuan Ding, Ying Yang, Xing Fu, Jianfeng Zhou, Chad M. McKee, Ruth J. Muschel, Robert P. Gale, Jane F. Apperley, Danmei Xu

**Affiliations:** 1 Department of Haematology, Tongji Hospital, Tongji Medical College, Huazhong University of Science and Technology, Hubei, China; 2 Department of Endocrinology, Tongji Hospital, Tongji Medical College, Huazhong University of Science and Technology, Hubei, China; 3 Gray Institute of Radiation Oncology and Biology, Medical Science Division, University of Oxford, Oxford, UK; 4 Department of Haematology, Hammersmith Hospital, Imperial College Healthcare NHS Trust, Imperial College London, Du Cane road, London, W12 0HS UK

Diffuse large B cell lymphoma (DLBCL) is the most common non-Hodgkin lymphoma (NHL) in adults^1^. One-third to half of people with DLBCL is not cured despite substantial therapy advance^2^. The variability of outcome is associated with prognostic scoring system such as the International Prognostic Index (IPI), which focus on biological features of the host and of the neoplastic cells^1^. However, considerable recent data suggest lymphoma microenvironment contributes to prognosis by affecting lymphoma cell survival and angiogenesis^3–5^. For example, gene chip from DLBCL tissues revealed a stromal signature that associated with favorable prognosis^5^. This signature is predominantly composed of genes encoding extracellular matrix (ECM) components and modifiers such as laminin, fibronectin, and collagen^5^. Those with a *stromal 2* pattern encoding angiogenesis-related genes are unfavorable^5^.

Matrix metalloproteinase-9 (MMP-9) regulates tumor microenvironment by degrading ECM and collagen type-IV, disrupting the physical barrier to facilitate cancer cell invasion^6^. *MMP9* is over-expressed in high-grade NHL, associated with an unfavorable prognosis in DLBCL^6,7^. SB3CT, a potent gelatinase inhibitor against MMP-9/MMP-2, inhibits angiogenesis, prevents formation of lymphatic vessels, and reduces the spread of lymphoma cells in a mouse T cell lymphoma model^8^. However, little is known of how MMP-9 regulates the lymphoma microenvironment.

In a prior study through a proteomic approach, we showed that protease nexin-1 (PN-1) is susceptible to MMP-9-mediated degradation in the prostate cancer microenvironment^9^. PN-1, a 43 kDa serine protease inhibitor, prevents several key micro-environmental proteases including urokinase plasminogen activator (uPA, encoded by *PLAU*), tissue plasminogen activator (tPA), thrombin, and plasmin^10,11^. High levels of MMP-9 and uPA are associated with disease progression and an unfavorable prognosis in several human solid cancers^11^. Although deregulation of PN-1 occurs in several solid cancers, the role if any, of PN-1 in hematological cancers is unknown. The coincidence of *MMP9* and *PLAU* gene found in the DLBCL *stromal 1* signature^5^ suggests a possible link between expression of these genes and the lymphoma microenvironment.

First, low levels of PN-1 were found in lymphoid tissues (lymph nodes and spleen) infiltrated with DLBCL cells compared with controls (Fig. 1a). High transcript levels of *MMP9* were found in the DLBLC-infiltrated tissues, consistent with prior reports and gene chip data^5^. In contrast, *PN1* transcript levels in DLBCL-infiltrated tissues were significantly lower compared with reactive lymph nodes (Fig. 1b). Interestingly, in contrast to the low transcript levels of *PN1* in the DLBCL-infiltrated bone marrow, bone marrow from subjects with DLBCL without bone marrow involvement had high levels of *PN1* transcripts (Fig. 1c). Thus, PN-1 levels appear reduced in B cell lymphoma.

**Fig. 1:**
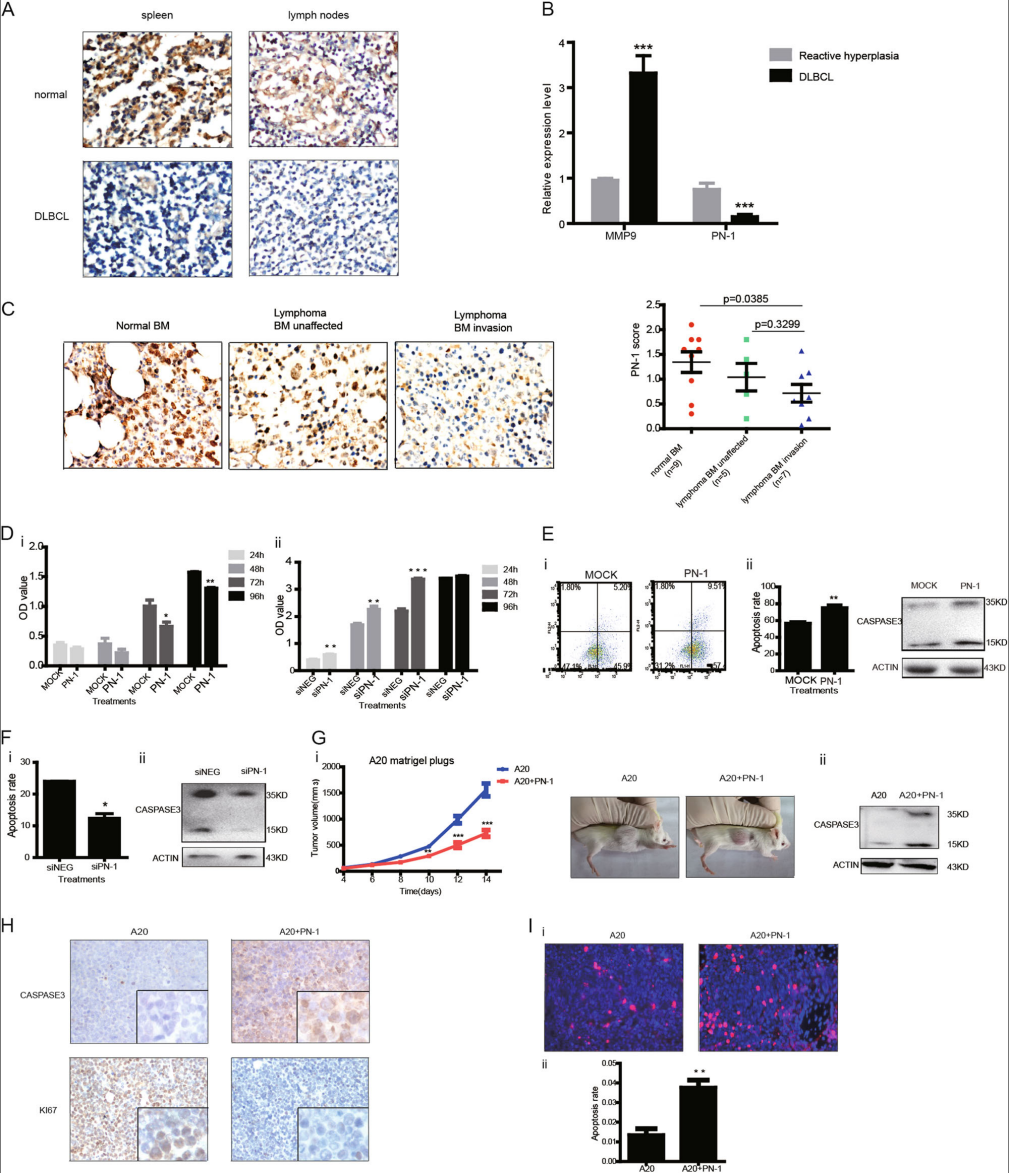
The expression pattern of PN-1 in DLBCL and its functional impact on lymphoma cell proliferation and apoptosis. **a** Immune histo-chemical staining (brown) of PN-1 in spleen and lymph nodes from normals or infiltrated with DLBCL cells. Blue stain represents haemotoxylin nuclear staining. **b** Quantitative real-time PCR for the expression of *MMP9* and *PN1* in lymph nodes hyperplasia (LN) and DLBCL. *N* = 6, ***P < 0.001. Student's *t* test was performed for statistical significance. **c** DAB staining of PN-1 (brown, panel i) and relative expression score (ii) in controls with a normal bone marrow (*N* = 9), persons with lymphoma with an involved (*N* = 7) or uninvolved bone marrow (*N* = 6). One-way ANOVA performed for statistical significance. **d** Proliferation of Raji cells (2 × 10^6^) was analyzed by using CCK-8 assays at 24, 48, 72, and 96 h after transfection with 2 μg of pcDNA3-*PN1* or control plasmid (i) or followed by treatment of 40 pmol siRNA *PN1* (si*PN1*) or control scrambled siRNA (siNEG) (*N* = 3; *t *test *P < 0.05; **P < 0.01; ***P < 0.001); **e** Apoptosis analysis was determined by Annexin-PI assay (i) and immune-blotting of cleavage of caspase-3 (15-kDa active band) (ii) in Raji cells transfected with 2 μg of pcDNA3-*PN1* or control plasmid (*N* = 4; *t *test **P < 0.01); **f** Raji cells transfected with siRNA *PN1* (si*PN1*) or control siRNA (siNEG) were assayed for apoptosis (*N* = 3; *t* test *P < 0.05); **g** Subcutaneous tumor volumes from Balb/c mice implanted with A20 cells (1 × 10^6^) mixed with Matrigel with or without PN-1 recombinant protein (10 μM). Subcutaneous tumor growth curve (i) (*N* = 5; *t *test, **P < 0.01; ***P < 0.001) and tumor volumes (ii); immune-blotting detecting caspase-3 cleavage (iii); **h** A20 xenografts with or without pre-treatment with PN-1 (10 μM) recombinant protein, DAB-stained (brown) for caspase-3 and Ki67. Blue stain represents hematoxylin nuclear staining. (**i**) in situ apoptotic assay using TUNEL. Positive cells (red stain) were counted from three microscopic fields and plotted (*N* = 4, *t* test, **P < 0.01). Nuclei are blue stained

To determine how PN-1 affects the biology of lymphoma cells, we examined endogenous *PN1* transcript levels in a number of human B cell lymphoma cell lines (Figure S1A). In contrast to low *PN1* expression, *MMP9* transcript levels were highest in Raji cells (derived from Burkitt lymphoma) (Figure S1B). In contrast, Wsunhl and Jeko cells (derived from follicular lymphoma and mantle cell lymphoma respectively) with lower *MMP9* transcript levels than Raji cells had high levels of *PN1* transcripts (Figure S1B), in keeping with a known regulation of PN-1 being a substrate target of MMP-9^9,12,13^. Adding exogenous PN-1 prevented proliferation of Raji cells and A20 cells (a mouse B cell lymphoma cell line) while knockdown of *PN1* expression by siRNA increased proliferation of these cells (Figs. 1di–ii and S2A). Adding exogenous *PN1* to Raji and A20 cells increased apoptosis determined by flow cytometry and by the caspase-3 assay (Figs. 1e and S2B). In contrast, apoptosis decreased in cells where *PN1* expression was reduced by siRNA (Fig. 1f).

Next, we developed a syngeneic B cell lymphoma mouse model by subcutaneous injection of A20 cells into Balb/c mice. Adding recombinant PN-1 protein (10 μM) to the Matrigel plug significantly delayed lymphoma growth (Fig. 1gi). Tumor volumes of mice treated with exogenous PN-1 protein after 12 days were 500 mm^3^ compared with 989 mm^3^ in controls (*p* < 0.001). Apoptosis rates in the tumor grafts were examined in situ using a TUNEL assay and/or immune blotting or immune-histochemistry for caspase-3 cleavage (Fig. 1gi). Adding PN-1-induced apoptosis while inhibiting proliferation as evidenced by markedly increased caspase-3 staining and decreased Ki67 staining (Fig. 1h).

The *SHH*-signaling pathway is activated in DLBCL and contributes to lymphoma cell survival and proliferation^14^. Over-expression of *PN1* resulted in a reduced transcription and protein expression of molecules in the SHH-signaling pathway including *SHH*, *GLI1*, and *PTCH1* in human Raji and mouse A20 cells (Figs. 2a and S2C). Conversely, *PN1* downregulation by siRNA was associated with increased expression of *SHH* and *GLI1* (Fig. 2b), indicating *PN1* expression inhibits *SHH* signaling in lymphoma cells. Here, we compared inhibitory effects of PN-1 on lymphoma cells with those of the *SHH* signaling inhibitor cyclopamine, which irreversibly binds SMO and inactivates SHH signaling. Raji cells treated with exogenous *PN1* or 10 μM cyclopamine can both prevent SHH-signaling transcription (Fig. 2c). There was more inhibition of SHH signaling when cells were treated with *PN1* plasmid transfection and cyclopamine compared with cyclopamine alone (Fig. 2d). Increasing concentrations of cyclopamine produced a dose-dependent decrease of mRNA levels of *GLI1* and *SHH* (Figure S3A). Adding PN-1 recombinant protein had a similar effect as cyclopamine in reducing *GLI1* and *SHH* expression in A20 cells (Figure S3B–C). At 48 and 72 h, cyclopamine was a stronger inhibitor of proliferation, whereas effects on apoptosis were similar (Fig. 2e). We also determined *SHH* and *GLI1* transcript levels in A20 cells treated with recombinant PN-1. Levels of *SHH* and *GLI1* transcripts were reduced by 96.8 and 86.8% after treatment with 100 ng/ml recombinant PN-1 (Fig. 2f). Apoptosis of A20 cells induced by recombinant PN-1 was similar to that induced by cyclopamine (Figure S3D).

**Fig. 2:**
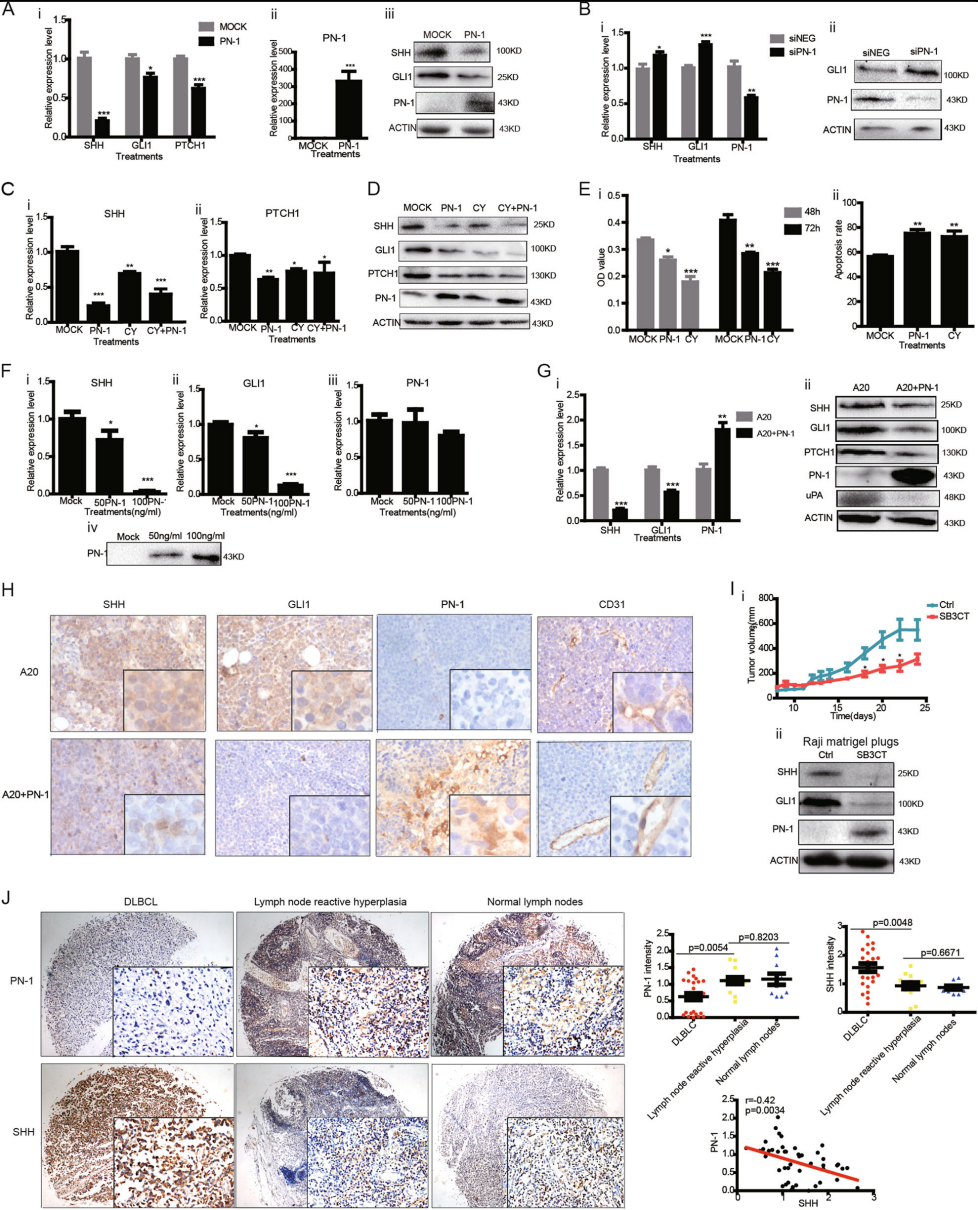
PN-1 inhibits SHH signaling and angiogenesis of B cell lymphoma. **a** Real-time PCR (i–ii) and immunoblotting (iii) were applied to determine the expression of *PN1*, *GLI1*, *PTCH1*, and *SMO* in Raji cells transfected with a *PN1* expression plasmid or empty vector (2 μg) for 24 h (*N* = 5, *t *test, *P < 0.05; ***P < 0.001). **b** Raji cells transfected with 40 pmol siRNA *PN1* (si*PN1*) or control siRNA (siNEG) were measured for *PN1*, *SHH*, or *GLI1* transcript levels using qRT-PCR (i) and protein levels by immune-blotting (ii) (*N* = 5; *t* test, *P < 0.05; **P < 0.01; ***P < 0.001). **c** Transcriptional levels of *SHH* (i), *PTCH1* (ii) in Raji cells transfected with pcDNA3-*PN1* plasmid (2 μg) or treated with a SMO inhibitor cyclopamine (20 μM) or both for 24 h (*N* = 6; one-way ANOVA; *P < 0.05; **P < 0.01; ***P < 0.001). **d** Immune-blotting of SHH-signaling pathway molecules and PN-1 from Raji cells treated as indicated above. **e** Cell proliferation (**i**) was analyzed by CCK-8 proliferation assay after 48 and 72 h culture of cells treated with *PN1* transfection (2 μg) or cyclopamine (20 μM); apoptosis (ii) was determined by flow cytometry via Annexin-PI staining at 24 h as indicated above (*N* = 4; one-way ANOVA *P < 0.05; **P < 0.01; ***P < 0.001). **f** A20 cells (2 × 10^6^) were treated with mouse PN-1 recombinant protein at 50 or 100 ng/ml and mRNA transcripts *SHH* (i), *GLI1* (ii), and *PN1*(iii) were measured by *q*-RT-PCR (*N* = 3; one-way ANOVA *P < 0.05; ***P < 0.001). Immune-blotting of PN-1 protein was performed using A20 cell conditioned medium (iv); **g** real-time PCR (i) for *PN1*, *SHH*, and *GLI1* in A20 xenografts with or without PN-1 (10 μM) (*N* = 5; *t* test, **P < 0.01; ***P < 0.001); A20 xenografts with or without PN-1 (10 μM) were blotted via immune blotting (ii). **h** immune-histochemistry (brown) for SHH-signaling cascades and PN-1 in A20 xenograft tumor. DAB-stained for the angiogenesis marker CD31 (brown). Blue stain is hemotoxylin nuclear staining; **i **(i) Subcutaneous tumor volumes from NOD/SCID mice implanted with Raji cells (5 × 10^6^) mixed with PBS or Matrigel with or without SB3CT (7.5 μM) were measured and plotted (*N* = 3; *t* test *P < 0.05). (ii) Immune blots of PN-1, SHH, and GLI1 in Raji Nod/SCID xenografts with or without SB3CT (7.5 μM). **j** DAB staining of PN-1 and SHH in human lymphoid tissues. Measurement of staining intensity compared with lymph nodes from normal (*N* = 10), lymph node reactive hyperplasia (*N* = 13), or DLBCL-infiltrated lymph nodes (*N* = 24). One-way ANOVA performed. Correlation between PN-1 and SHH calculated using Spearman *r* value

SHH protein strongly expresses in DLBCL cells but not in less aggressive lymphomas, CLL and normal germinal center B cells^14^. We next studied the activation state of *SHH* signaling in vivo in a syngeneic B cell lymphoma model described in Fig. 1g. Twelve days of PN-1 treatment markedly reduced mRNA levels of *GLI1* and *SHH* (Fig. 2gi). Angiogenesis is associated with aggressive subtypes of B cell lymphomas^15^. To determine the impact of PN-1 on angiogenesis, we used CD31 staining in mice grafted with A20 lymphoma cells. Lymphomas grown with exogenous PN-1 recombination protein had lower vascular density (Figs. 2h and S4). Residual blood vessels had larger diameters than controls. There was also less in situ protein levels of *SHH*-signaling molecules in tissues treated with PN-1 shown by immune blotting of whole tissue lysates and by immune-histochemistry (Fig. 2gii–h). Thus, PN-1 appears having an anti-angiogenic role in B cell lymphoma.

Interestingly, *MMP9* expression and SHH signaling are both known of regulating the growth and survival of diverse NHL cells^5,7,15^. Our data suggest that PN1 might be a key player who connects these two important molecules. We subcutaneously injected Raji cells into NOD/SCID mice. Adding 7.5 μM SB3CT to the Matrigel plug inhibited growth of the Raji cells compared with control Matrigel plugs (Fig. 2i–i). At 24 d the tumor volume of treated mice were 260mm^3^ versus 550 mm^3^ in controls (p < 0.05; Fig. 2i–i). SB3CT treatment resulted in increased levels of PN-1 and reduced levels of SHH and GLI (Fig. 2i–ii). These data suggest that PN-1 inhibition of *SHH* signaling is governed by MMP-9, presumably attributed to increased stability of PN-1 upon chemical inhibition of MMP-9 activity by SB3CT.

Finally, because of the heterogeneity of DLBCL, we determined the levels of SHH and PN-1 in normal lymph nodes, reactive lymph nodes and lymph nodes infiltrated with DLBCL cells. SHH protein localizes predominantly to the cytoplasm of the lymphoma cells but sparse in the microenvironment (Fig. 2j). PN-1 expression was evident in the stroma and vessels of normal and reactive lymph nodes but less so in lymph nodes infiltrated by DLBCL cells (Fig. 2j). There was an inverse correlation between PN-1 and SHH levels in tissues infiltrated by DLBCL cells (*r* = −0.42; *p* = 0.0034). These data suggest lymph nodes infiltrated by DLBCL cells had variable expression of both proteins, whereas normal lymph nodes were less variable. This finding is not surprising in view of the considerable heterogeneity of DLBCL. Gene chip analyses from 414 newly diagnosed cases of DLBCL revealed *MMP9* and *PLAU* in the stromal signature, suggesting genes encoding ECM proteases such as *MMP9* might play critical roles on progression of B cell lymphoma^5^. However, considerable data suggest a striking heterogeneity in gene expression profiles and clinical outcomes of DLBCL, posing a challenge for clinical management. Consequently, molecular regulation of MMP-9, PN-1, and SHH may be different across DLBCL subtypes. SHH is produced by dendritic cells in the germinal center (GC) protecting germinal center B cell from apoptosis^15^. DLBCL cells secrete and respond to endogenous SHH ligands, implicating an autocrine SHH-signaling loop^14^. Our data from immune histo-chemical staining supports this concept.

Taken together, we present data supporting a new pathway by which *PN1* expression regulates lymphoma cell survival by inhibiting SHH signaling, an important survival signaling in the growth of B cell lymphomas. PN-1 regulates SHH signaling by reducing *SHH* transcripts and downstream effectors and by inhibiting angiogenesis. Such a regulation is also reflected by the inverse correlation between PN-1 and SHH proteins demonstrated in human DLBCL tissue microarray samples. The crosstalk between MMP-9, PN-1, and SHH signaling supports an inhibitory role of PN-1 in B cell lymphoma progression. MMP-9 and PN-1 may serve as potential molecular targets for combination therapies in DLBCL, in particular for those lymphomas with high expression of *MMP9*.

## Electronic supplementary material

Supplementary Table

supplemental information
