# Supplementary material for: Protease nexin-1 prevents growth of human B cell lymphoma via inhibition of sonic hedgehog signaling
Source: Blood Cancer J. 2018 Feb 26;8(2):24. doi: 10.1038/s41408-018-0063-x (PMC5827524; doi:10.1038/s41408-018-0063-x)
Supplement: Supplementary file 2 — supplemental information [file 41408_2018_63_MOESM2_ESM.doc]

**Supplemental information**

**Material and methods**

**Animals**. Animal experiments were performed in accordance with the guidelines by the Animal Care Committee of Tongji Medical College. Male Balb/c and NOD-SCID mice ( 6 weeks old), purchased from Fukang Biological Technology (Beijing, China), were randomized to distinct treatment groups, sacrificed at different time points and their tissues harvested and rinsed with PBS before stored at −80°C. Proteins were extracted from frozen tissues by standard techniques^9^ and concentrations determined by bicinchoninic acid assay (BCA; Thermo-Fisher, USA).

**Plasmids and siRNA**. pcDNA3-*PN1* plasmid, siRNA against *PN1* and siRNA negative control oligonucleotides were described.^9^ Raji and A20 cells were seeded into a 12-well plates at a concentration of 1 ×10^6^/ml for 24 h before transfection with 2 μg *PN1* plasmids or 40 pmol siRNA against *PN1* using the Lonza 4D-Nuclefector or Nucleofector™ Kits respectively (Amaxa Cell Line Nucleofector Kit SG, #V4XC3024; Amaxa Cell Line Nucleofector Kit V, #VCA-1003, Switzerland).

**Cell cultures.** Raji, Jeko, Sudhl6, Wsu-nhl6 and A20 cell lines were purchased from ATCC (Manassas, VA, USA) and maintained in RPMI-1640 supplemented with 10% fetal bovine serum at 37°C with 5% CO_2_. Cells were periodically examined to rule out mycoplasma infection. Cells were incubated for 24 h in serum-free medium with human recombinant protein PN-1 (R&D, #2980-PI-010, USA), SHH signalling inhibitor cyclopamine (Sigma #C4116, USA) or MMP-9 inhibitor SB3CT (Calbiochem, #444272, USA). Conditioned media were collected and concentrated using Amicon Ultra centrifugal filter units (10K cutoff; Millipore, Billerica, MA, USA).

**Cell proliferation assay**. *in vitro* cell proliferation was assayed by Cell Counting Kit-8 (CCK-8; Beyotime, #C0039, China). Briefly, 1×10^4^cells were seeded onto each well of a 96-well plate. 10 μl CCK-8 solution was added to each well at the times 0 and 24, 48, 72 and 96 h followed by 4h more incubation. Absorbance at 450 nm was measured using a Microplate Reader (Bio-Rad, USA).

**Flow cytometry**. 1×10^5^ cells were seeded onto each well of a 24-well plate for 24h and double stained using an Annexin V-FITC. Apoptosis detection kit (KeyGen BioTech, #KGA017, China) following the manufacturer’s instructions. Apoptosis was measured using a flow-cytometry (BD FACSCalibur® Flow Cytometer, USA) and data analyzed using FlowJo™ 7.6 software.

**Immuno-blotting, angiogenesis array and ELISA.** Whole cell lysates were prepared as described.^12^ Conditioned medium was concentrated and normalized for cell numbers and intracellular protein concentrations as described.^12^ Immuno-blotting was performed as described.^9^ Primary antibodies included: (1) anti-human PN-1 (R&D Systems, #AF2175, #AF2980, USA; Protein Tech Group, #11303-I-AP, China); (2) anti-mouse PN-1 (Santa Cruz Biotechnology, #sc817817, USA); (3) anti-human and mouse GLI1 (R&D Systems, #MAB3324, USA); (4) anti-human and mouse SHH (Santa Cruz Biotechnology, #sc9024, USA); (5) and anti-human and mouse β–actin (affbiotech,#T0022, USA), caspase3 (Servicebio, #GB13009, China). (6) anti-mouse uPA (ABGENT, #AP20302b, USA). Electro-chemo-luminescence (ECL) signal was detected by a SuperSignal West Pico kit (ThermoFisher Scientific #35065, USA). Conditioned medium was collected from Raji cell incubated with PN-1 recombinant protein (100 ng/ml) for 24 h. Human angiogenesis array (RayBiotech, #AAH-ANG-G1000, USA) was performed according to the manufacturer’s instructions. Specific enzyme-linked immune-absorbent assay (ELISA) kits for VEGF (BBI life sciences, #D151116, China), endostanin (Raybiotech, #ELH-Endostatin, USA) and TNF-alpha (Multi Science, #70EK1821, China) were used to measure cytokine levels according to the manufacturer’s instructions. Cytokine concentration was normalized to cell numbers in the well when the supernatant was collected.

**TUNEL assay and immune histo-chemistry**.

Frozen sections of mouse subcutaneous lymphoma xenografts were stained by terminal deoxynucleotidyl transferase dUTP nick-end-labeling (TUNEL) assay kit (Chemicon International, #S7165, USA) as described.^11^ Human tissue microarrays (TMA; BioMax, #LY800a, USA) were used to stain PN-1 and SHH levels (10 normal lymph nodes, 13 lymph nodes with reactive hyperplasia and 24 lymph nodes infiltrated with DLBCL). TMA staining was assessed using a combination of intensity and percent stained cells as described.^13^ We used tissues fixed in 4% paraformaldehyde or embedded in optimal cutting temperature compound (OCT) and sliced into 10 μm thick frozen sections. Samples were stained with specific antibodies against PN-1, SHH (Santa Cruz Biotechnology, #sc817817, #sc9024, USA), GLI1 (R&D Systems, #MAB3324, USA) and caspase-3, ki-67, CD31 (Servicebio, #GB13009, #GB13030-2, #GB14008, China) following antigen retrieval. Pre-diluted secondary antibody (Servicebio) was added to the slides for 1 h followed by impact DAB (Servicebio, #G1003-2, China) staining. Slides were counter-stained with haematoxylin and mounted with neutral gum. Samples were evaluated microscopically and photographs taken using a 10× eyepiece. Tissue microarrays were imaged at 100× and 400× magnifications.

**RNA isolation and quantitative RT-PCR.**

RNA from cell pellets or subcutaneous tumors was extracted by Trizol reagent and RNA concentration measured by a Nano-Drop 1000 spectrophotometer (Thermo). cDNA was synthesized using a RevertAid First Strand cDNA Synthesis Ki™ (Thermo Scientific, #K1622, USA). Quantitative PCR was performed on a Bio-Rad Thermocycler using SYBR Green real Time PCR method. Samples were tested in triplicate. Amplified products were normalized against *GAPDH* or *ACTB* (β-Actin) transcripts. Primer sequences are listed in the Supplement Table 1.

**Tumour assay.**

Balb/c mice were injected subcutaneously with 1 ×10^6^ mouse lymphoma A20 cells in 50μl of serum-free media and 50μl of Matrigel. 10μM of PN-1 recombinant protein (R&D Systems, #2175-PI-010, USA) was added where indicated. NOD/SCID mice were injected subcutaneously with 5×10^6^ Raji cells in 50μl of serum-free media mixed with 50μl of Matrigel. 7.5 μM SB3CT was added where indicated. Tumor volumes were determined by vernier caliper measurement reported in mm^3^ using the W^2^ × L/2 equation where W indicates width and L, length.^13^

**Statistics**. Statistical comparisons were determined using Prism 5 GraphPad™ software. Data represent the mean ± SEM. A P-value ≤0.05 was considered significant.

**Supplementary Figures**


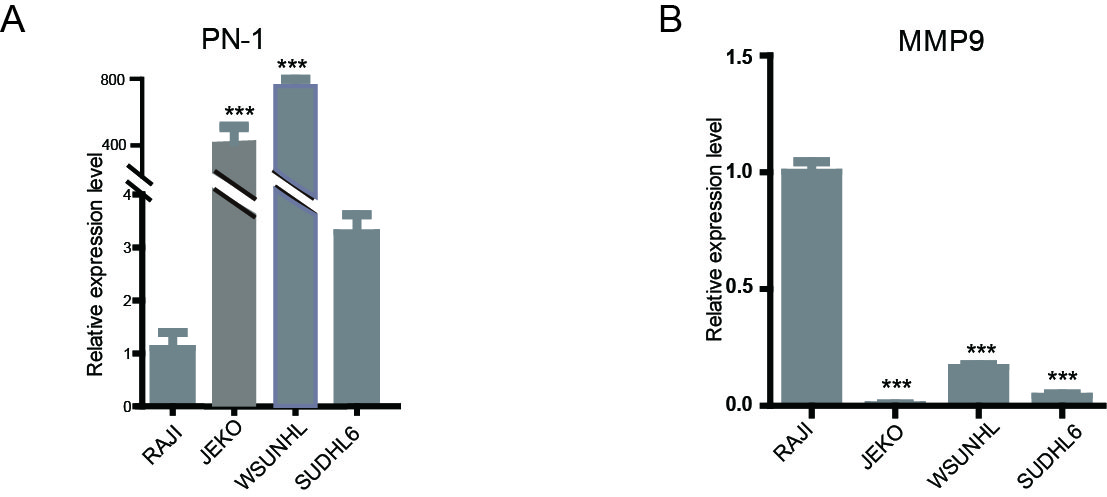


**Figure S1.**  Relative transcriptional levels of *PN1* (A) and *MMP9* (B) determined by the quantitative real-time PCR in different B-cell lymphoma lines including RAJI, JEKO, WSUNHL, and SUDHL6 (N=3, one-way ANOVA; ***P<0.001).


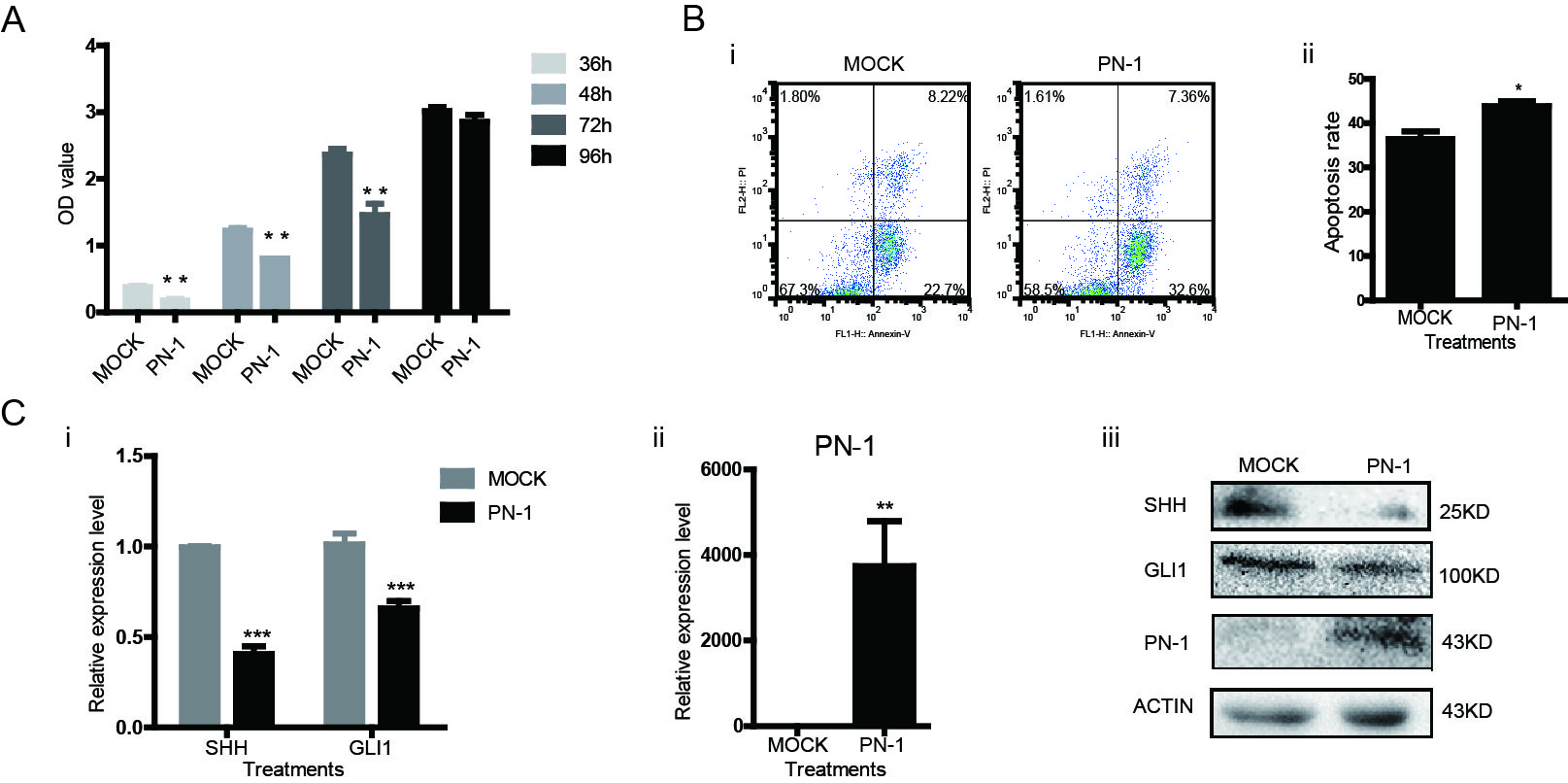


**Figure S2. PN-1 decreases the SHH-signalling cascade, inhibits proliferation and promotes apoptosis of mouse DLBCL cells.** (A-B) A20 cells (2x10^6^) transfected with 2 μg of mock or *PN1* expressing vector and proliferation analysis *via* CCK8 kit (A); apoptosis analysis using the Annexin-PI assay (B); (C) The transcript levels of *PN1*, *SHH*, *GLI1* using qRT-PCR (i-ii) and protein levels using immune blotting (iii) (N=3; *t-*test *P<0.05; **P<0.01; ***P<0.001).


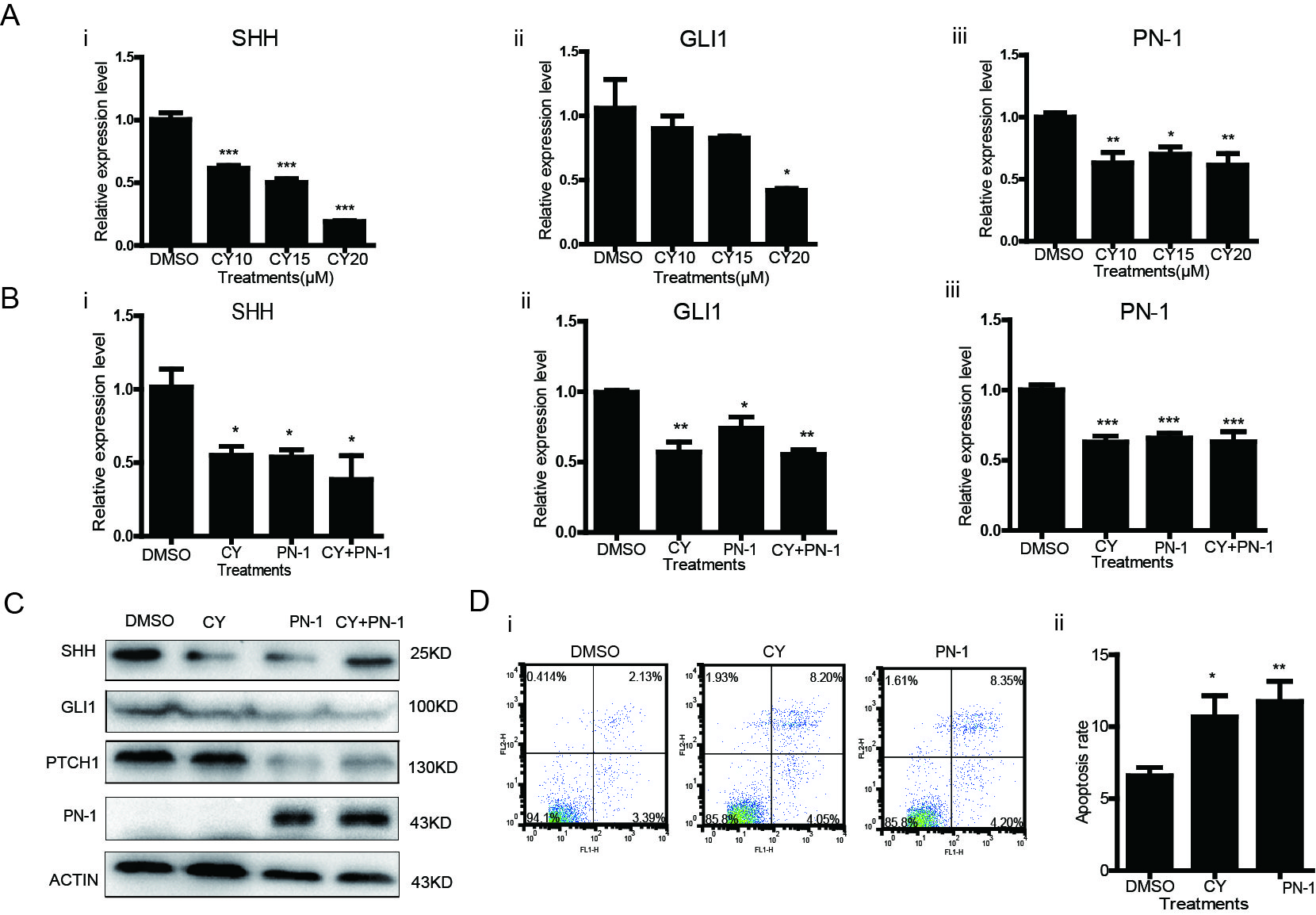


**Figure S3. PN-1 inhibits SHH signaling in mouse DLBCL cells.** (A) Real-time PCR for *GLI1*, *SHH*, *PN1* in A20 cells (2x10^6^) treated with cyclopamine at concentrations of 10, 15 and 20 μmol/l for 24 h. Transcript levels (B) and protein levels (C) of SHH-signaling pathway molecules and PN-1 in A20 cells treated with 100 ng/ml of recombinant PN-1 proteins, cyclopamine (20 μM) alone or both for 24 h; (D ) Apoptosis of A20 cells treated as indicated was measured and plotted (ii) (N=3; one-way ANOVA *P<0.05; **P<0.01; ***P<0.001).


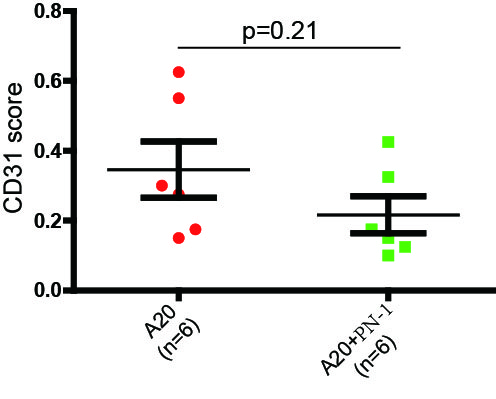


**Figure S4.** The relative expression score (ii) in A20 xenografts with or without PN-1 (10 μM) were DAB-stained for the angiogenesis marker CD31 (brown). (N=6; one-way ANOVA)
